# Study of influence of the glutamatergic concentration of [^18^F]FPEB binding to metabotropic glutamate receptor subtype 5 with N-acetylcysteine challenge in rats and SRM/PET study in human healthy volunteers

**DOI:** 10.1038/s41398-020-01152-2

**Published:** 2021-01-20

**Authors:** Anne-Claire Dupont, Sophie Serrière, Laurent Barantin, Johnny Vercouillie, Clovis Tauber, Valérie Gissot, Sylvie Bodard, Gabrielle Chicheri, Sylvie Chalon, Pr Frédérique Bonnet-Brilhault, Pr Maria-Joao Santiago-Ribeiro, Nicolas Arlicot

**Affiliations:** 1 Service de Radiopharmacie, CHRU de Tours, 37044 Tours, France; 2 UMR 1253, iBrain, Université de Tours, Inserm, Tours, France; 3 INSERM CIC 1415, CHRU de Tours, Tours, France; 4 Centre Universitaire de Pédopsychiatrie, CHRU de Tours, 37044 Tours, France; 5 Service de Médecine Nucléaire, CHRU de Tours, 37044 Tours, France

**Keywords:** Molecular neuroscience, Diagnostic markers

## Abstract

Altered glutamate signaling is thought to be involved in a myriad of psychiatric disorders. Positron emission tomography (PET) imaging with [^18^F]FPEB allows assessing dynamic changes in metabotropic glutamate receptor 5 (mGluR5) availability underlying neuropathological conditions. The influence of endogenous glutamatergic levels into receptor binding has not been well established yet. The purpose of this study was to explore the [^18^F]FPEB binding regarding to physiological fluctuations or acute changes of glutamate synaptic concentrations by a translational approach; a PET/MRS imaging study in 12 healthy human volunteers combined to a PET imaging after an N-acetylcysteine (NAc) pharmacological challenge in rodents. No significant differences were observed with small-animal PET in the test and retest conditions on the one hand and the NAc condition on the other hand for any regions. To test for an interaction of mGuR5 density and glutamatergic concentrations in healthy subjects, we correlated the [^18^F]FPEB BP_ND_ with Glu/Cr, Gln/Cr, Glx/Cr ratios in the anterior cingulate cortex VOI; respectively, no significance correlation has been revealed (Glu/Cr: *r* = 0.51, *p* = 0.09; Gln/Cr: *r* = −0.46, *p* = 0.13; Glx/Cr: *r* = −0.035, *p* = 0.92).These data suggest that the in vivo binding of [^18^F]FPEB to an allosteric site of the mGluR5 is not modulated by endogenous glutamate in vivo. Thus, [^18^F]FPEB appears unable to measure acute fluctuations in endogenous levels of glutamate.

## Introduction

Glutamate, the most abundant neurotransmitter in the central nervous system (CNS), is primarily involved in the synaptic excitatory activity^1^. Thus, it intervenes in several brain functions such as memory, learning, behavior, and movement^2^. However, when present at an excessive concentration, glutamate may contribute to neuronal dysfunction. Indeed, glutamate is only removed from the synaptic cleft thanks to two major astrocytic transporters, namely GLT-1 (EAAT2) and GLAST (EAAT1), regulating receptor’s excitability^3^. Alterations of this glutamatergic homeostasis may lead to various neurological and psychiatric disorders^4–9^. Due to their substantial role in brain functions, elucidating the mechanism underlying glutamate receptor’s action would clarify glutamate’s role in physiological and pathological conditions. There are two main classes of glutamate receptors: (i) the ionotropic receptors *(α-amino-3-hydroxy-5-methyl-4-isoxazolepropionic acid receptor (AMPA), Kainate receptor and N-methyl-d-aspartate receptor (NMDA))*, involved in the fast-acting excitatory effects and, (ii) the metabotropic receptors (mGluR), mainly implicated in the modulation of the glutamatergic neurotransmission. Those metabotropic glutamate receptors are classified into three subtypes, based on both their structure and functions: group I (mGluR1 and mGluR5), group II (mGluR2 and mGluR3), and group III (mGluR4, and mGluR_6–8_). The mGluR belong to the G-protein coupled receptors (GPCRs), which represent the largest class of drug targets, accounting for more than 40% of marketed drugs^10^. Among them, metabotropic glutamate receptors subtype 5 (mGluR5) have been demonstrated to play crucial roles in synaptic plasticity and neuronal development^11,12^, and have especially emerged as a promising target for a broad range of neurological disorders. Widely expressed and mainly postsynaptic throughout the cerebral cortex, corpus striatum, hippocampus, olfactory bulb, caudate nucleus, nucleus accumbens but also in non-neuronal cells including astrocytes and microglia, mGluR5 have been implicated in the pathogenesis of numerous psychiatric, neurological, and neurodevelopmental disorders including schizophrenia, addiction, anxiety, depression, PD, fragile X syndrome (FXS), or autism spectrum disorders (ASD)^2,13–17^ Firstly, drug development programs targeting mGluR5 were interested in the orthosteric site of the receptor. However, recent findings have clearly demonstrated that allosteric modulators that interact with binding sites distinct from the endogenous agonist glutamate had better potential. Indeed, they exhibit numerous advantages including higher subtype selectivity, better blood‐brain-barrier penetration, and absence of desensitization which is caused by orthosteric ligands after repeated administrations^18,19^. An allosteric modulator can potentiate glutamate response in the case of positive allosteric modulator (PAM) or reduce glutamate response in the case of negative allosteric modulator (NAM)^18^. NAMs (e.g., basimglurant, mavoglurant, and dipraglurant) were the first mGluR5 allosteric modulators evaluated into clinical trials^19–22^.

In relation to these therapeutic trials, intense efforts have been made to develop biomarkers and companion tests, including for noninvasive techniques allowing glutamatergic transmission imaging.

Positron emission tomography (PET) utilizes positron emitter labeled compound to provide valuable information about the target availability under normal and pathological conditions, and then allow evaluating drug intervention. PET can be used to quantify the receptors, transporters, or enzymes expressed at the level of nanomolar concentration in the living tissues. No PET tracer targeting mGluR’s orthosteric sites has been developed, since the high concentration of endogenous glutamate would dramatically compete with very low mass concentration of PET radiotracer^23^. Hence, several radiotracers targeting mGluR5 allosteric binding sites have been proposed to investigate in vivo glutamate neurotransmission by PET. The first suitable tracer for both preclinical and clinical use, namely [^11^C]ABP688, was developed in 2006^24^. Despite its favorable characteristics, its major limitation remains the short half-life of its radioisotope (about 20 min), limiting its use to centers with a cyclotron on site. Therefore, and to get around this drawback, two fluorinated radiotracers have recently been developed: [^18^F]PSS232 and [^18^F]FPEB. [^18^F]FPEB (3-[(18)F]fluoro-5-(2-pyridinylethynyl)benzonitrile) is a negative modulator of the mGluR5 allosteric sites with an excellent pharmacological profile with a human mGluR5 Ca^2+^ Ic_50_ flux of 0.66 nM, a *K*_i_ in rats of 0.20 nM, and a log*P* value of 2.8^25^. Thanks to its favorable reversible kinetics, a high specificity, and the absence of brain radiometabolites^26^, [^18^F]FPEB has been widely used to study in vivo dysfunctional glutamate transmission associated with neuropsychiatric including Parkinson’s disease (PD)^27^, major depressive disorder^28^, addictions (alcohol)^29^, and autism^30^.

The lack of sleep^31^ or smoking or a treatment by glutamate modulators^32–36^ could disturb glutamate balance and knowledge is lacking as to whether these endogenous fluctuations could hold sway over the mGluR5 allosteric binding. Numerous studies with [^11^C]ABP688 or [^18^F]PSS232 radiotracers based on pharmacological challenges that modulate endogenous glutamate levels attempted to provide insight into glutamate receptor availability for several PET mGluR5 radioligands^37–40^, but only one investigated whether [^18^F]FPEB could detect ketamine-induced changes in mGluR5 in healthy subjects^41^. Conflicting results were highlighted regarding both tracers and animal species. One PET study showed that pharmacological challenge with *N*-acetylcysteine (NAc), known to indirectly increase extrasynaptic glutamate release through activation of the cystine-glutamate antiporter (xc-)^32^, produced a decrease in the binding of [^11^C]ABP688 in baboons^42^. In contrast, repeated studies in both rats^39^ and Rhesus monkeys^40^ showed no effect of pharmacological challenge on [^11^C]ABP688 binding. Therefore, assessing the [^18^F]FPEB binding behavior with glutamate shift concentration seems to be valuable regarding its large use in clinical trials. The purpose of the present study was therefore to explore the sensitivity of [^18^F]FPEB binding to fluctuations of glutamate synaptic concentrations using a translational approach. We firstly used a combined PET/magnetic resonance spectroscopy (MRS) approach in healthy human volunteers. To this end, we analyzed glutamate (Glu), glutamine (Gln) and glutamate + glutamine (Glx) levels using ^1^H-MRS and receptor binding of [^18^F]FPEB to mGluR5. In a second step, regarding interspecies discrepancies observed with [^11^C]ABP688 or [^18^F]PSS232, we performed pharmacological challenge in rodents using NAc^32^.

## Materials and methods

### [^18^F]FPEB radiosynthesis

[^18^F]FPEB was labeled with fluorine 18 via direct nucleophilic substitution from its corresponding bromo-analog using a TracerLab FX_F-N_ chemistry module, and according to slight modifications of previously reported procedures for onsite radiopharmaceuticals productions^43^. [^18^F]FPEB was demonstrated to meet all quality control criteria for a PET radiotracer. Our translational approach required 12 [^18^F]FPEB productions for the preclinical challenge study and 12 for the clinical PET study. [^18^F]FPEB was obtained with a mean molar activity at the end-of-synthesis of 148 GBq/µmol and 118 GBq/µmol and, with a radiochemical yield of 9 and 6%, respectively.

### Preclinical evaluation of [^18^F]FPEB binding after N-acetylcysteine challenge in rats

#### Animals

All procedures were conducted in accordance with the European Community Council Directive 2010/63/EU for laboratory animal care and the experimental protocol was validated by the Regional Ethical Committee (Authorization N°10552101). Experiments were carried out on adult male Sprague–Dawley rats (Charles River laboratories) weighing 366 ± 31 g at the beginning of experiments. Animals were housed in groups of two per cage in a temperature (21 ± 1 °C) and humidity (55 ± 5%) controlled environment under a 12-h light/dark cycle, with food and water available ad libitum. An acclimatization period of five days was observed between rat arrival and the experiment outset.

#### Study design, PET imaging, and analysis

**Fig. 1:**
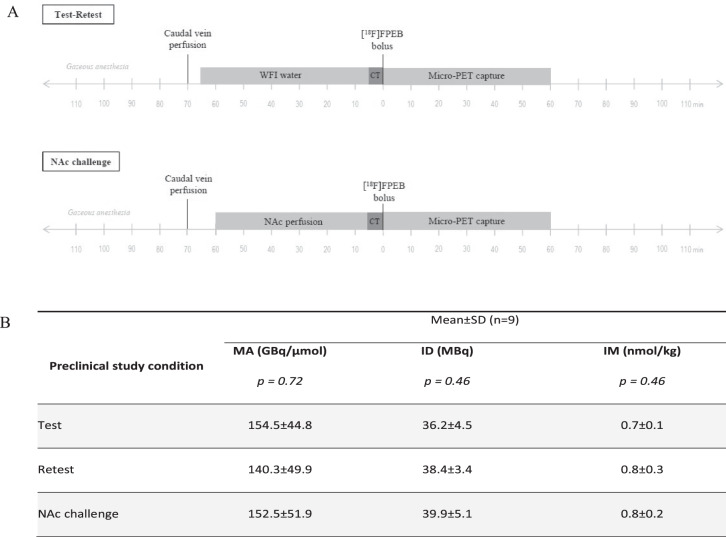
Preclinical evaluation of [^18^F]FPEB binding after NAc challenge. **A** Preclinical NAc challenge study design: rats received either a water for injection (WFI) or a NAc (50mg/kg) tail injection 60 minutes before the scan, a 5 minutes transmission scan (CT) preceded the 61 minutes PET emission scan. Each animal was its own control, so that it underwent 3 imaging sequences for the whole protocol (test, retest and NAc challenge). **B** Mean [^18^F]FPEB Molar Activity (MA), mean injected Dose (ID) and Injected Mass (IM) per preclinical study condition.

The study design is exposed in Fig. 1A. PET imaging of mGluR5 with [^18^F]FPEB was performed in male Sprague–Dawley rats (*n* = 9). Anesthesia was induced using isoflurane (Aerrane®, Baxter, France), at 3.5% in O_2_ and maintained at 1.5–2% during scanning. After catheterization into the tail, rats were scanned on separate days following a random order, according to the protocol described by Wyckhuys et al.^39^. For the NAc challenge, animals received a 60 min tail vein infusion of NAc (Hidonac 200 mg/mL®, Zambon France 50 mg/kg/h; 7.5 mg/mL). For test-retest experiments, rats were perfused with the vehicle of the NAc (injectable water, Lavoisier) 60 min before the scan. Subsequently, animals were submitted to PET imaging. PET acquisitions of 61 min with [^18^F]FPEB were performed on a microPET eXplore VISTA-CT® system (GE Healthcare, France), which has an effective axial field of view (FOV) of 4.8/6.7 cm, a spatial resolution of less than 2 mm, and a sensitivity above 2.5% in the whole FOV. For imaging, each rat was placed on a thermoregulated bed (Minerve, France) in the prone position with a nose cone. The brain was positioned on the center of the FOV. Before PET acquisition, 5 min computed tomography (CT) scan was acquired for attenuation correction. A bolus injection of 37 ± 3 MBq/300 g body weight of [^18^F]FPEB in saline was administered into the tail vein, 1 min after the beginning of the PET acquisition. PET experiments were carried out using microdose referring to the mass amount of the substance injected (<100 µg)^44^ and means of injected radioactivity were 36 ± 4 MBq, 38 ± 3 MBq, and 40 ± 5 MBq for test, retest and challenge, respectively (Fig. 1B). The absence of correlation between SUVs and this injected mass strongly suggests that PET acquisitions were performed with a tracer range of [^18^F]FPEB. The PET list-mode scans were rebinned into 27 frames (1 × 60 s before injection of the tracer followed by 3 × 10 s, 3 × 20 s, 7 × 30 s, 4 × 150 s, 9 × 300 s). Each PET scan was corrected for random, scatter, and attenuation, and the images were reconstructed using a 2D OSEM algorithm (GE Healthcare, France) into voxels of 0.3875 × 0.3875 × 0.775 mm^3^. A partial volume effect correction was applied on all images, which were co-registered in a single interpolation to the Schiffer rat brain MRI template as described in Serriere et al.^45^. All images were analyzed using PMOD® (version 3.403; PMOD Technologies, Zurich, Switzerland). The tissue radioactivity values of brain regions were decay-corrected and normalized to the injected radioactivity, and body weight resulting in standardized uptake values (SUVs). In this study, the standard uptake value ratio (SUVr) was used as quantitative criterions. All SUVr were calculated using the cerebellum as the reference region^46^. The PET images for the regions of interest (ROIs) of caudate putamen, amygdala, thalamus, cingulate, frontal cortex, and cerebellum were analyzed. For calculation of the time-activity curves, regions were defined on the rat MRI T2 template.

### Clinical ^1^H-MRS/PET study in healthy human subjects

#### Subjects

Healthy male volunteers were assessed by medical interview, questionnaires, and measurements of vital signs; key exclusion criteria as follows: (i) known psychiatric or neurological condition, (ii) active and former smoking, and (iii) drug or alcohol misuse (iv) medication intake interfering with glutamatergic transmission. Applied questionnaires included the autism spectrum quotient, empathy quotient, and the Mini International Neuropsychiatric Interview. All subjects were screened for neuropsychiatric and other medical disorders and were subjected to a physical examination, and structural brain MR imaging. Fourteen subjects were screened but only twelve completed both PET and ^1^H-MRS scans successfully. The French National Agency for Medicines and Health Products Safety (ref. number 150583A-12) and the Ethics Committee approved the study. It was conducted in line with the latest version of the World Medical Association Declaration of Helsinki. All subjects provided written informed consent prior to inclusion.

#### In vivo MRS acquisition and image analysis

All subjects were evaluated with 3 T routine brain MRI scans (Verio®, Siemens, Germany), and proton MR Spectroscopy (MRS) using a 12 channels head coil. The protocol consisted of sagittal T1-weighted and axial T2-weighted spin-echo images, followed by MRS. T1-weighted MRI scans were acquired for coregistration with PET images. Following automatic shimming to optimize field homogeneity across the voxel, single voxel Point RESolved (PRESS) ^1^H-MR spectra were acquired from a 33 × 20 × 9 mm^3^ voxel of interest (5.94 mL) positioned in the ventral and dorsal parts of the anterior cingulate cortex (ACC right and left). The voxel location covered the maximum amount of gray matter and the minimal amount of cerebral fluid to avoid contact with the macromolecules of the brain surfaces, which contaminate MRS spectra. The ACC was chosen due to its involvement in high-order integration processes (e.g., social interaction, language). MR spectra were acquired with an echo time (TE) of 35 ms, a repetition time (TR) of 2000 ms, 1024 points, 100 averages). This acquisition was followed by ten averages of unsuppressed water with the same acquisition parameters and localization to quantify metabolites according to water peak. Glu, Gln, and Glx levels were quantified both as water-scaled concentrations and as ratios to the composite creatine peak (Cr + PCr) (Glu/Cr, Gln/Cr, and Glx/Cr ratios) from MRS data.

Spectra were fitted with LCModel® (Version 6.3, Stephen Provencher, Canada), after eddy-current correction and water-scaling. Quantification reliability and spectra quality were assessed using Cramer-Rao lower bounds (CRLB) less than 25%. As an additional criterion for good spectral quality, metabolites levels were analyzed when the signal to noise ratio SNR was superior at 3 and the full width half maximum (FWHM) <0.1 ppm.

#### PET acquisition and image analysis

After a CT acquisition (40 mAs, 80kv) for PET attenuation purposes, [^18^F]FPEB (257 ± 32 MBq) was administered intravenously as a bolus and emission data was promptly collected in list mode for 59 min with an Ingenuity TF64 Time-of-Flight tomograph® (Philips, USA). List mode data were reconstructed to 31 frames (6 × 10 s, 8 × 30 s, 4 × 1 min, 5 × 2 min, and 8 × 5 min). Acquisition data were reconstructed with the standard package included with the system (PET view software-Philips Medical Systems). PET sinograms were corrected for tissue attenuation, decay, scatter, and random radiation, and then they were reconstructed using a 3D iterative RAMLA algorithm in voxels of 2 × 2 × 2 mm^3^. PET data were analyzed using PMOD software (version 3.4®; PMOD Technologies Ltd.). All PET images were first coregistered to subject’s T1 3D MRI. A volume-of-interest (VOI) analysis was performed using the N30R83 Hammers probalistic atlas, allowing for an observer-independent delineation of neuroanatomical regions^47^. The time activity curves (TAC) of the VOI were analyzed to calculate the non-displaceable binding potential (BPND) using the simplified reference tissue model 2 (SRTM2), with cerebellar white matter as the reference region^48^.

### Statistical analysis

For challenge preclinical PET imaging studies, results were expressed as mean ± standard deviation (SD). Statistical comparisons of test/retest small-animal PET datasets were performed using Wilcoxon signed-rank tests. Comparison between the three groups (test, retest, and challenge) was conducted by Friedman two-way analysis. Differences were considered statistically significant at *p* values < 0.05. To assess the association between [^18^F]FPEB BP_ND_ and Glu, Gln, Glx ratios in the ACC, Spearman’s rank correlation coefficients were calculated for univariate correlation analysis and the significance level was set at *p* < 0.05 (two-tailed).

## Results

### [^18^F]FPEB preclinical study after N-acetylcysteine challenge in rats

**Fig. 2:**
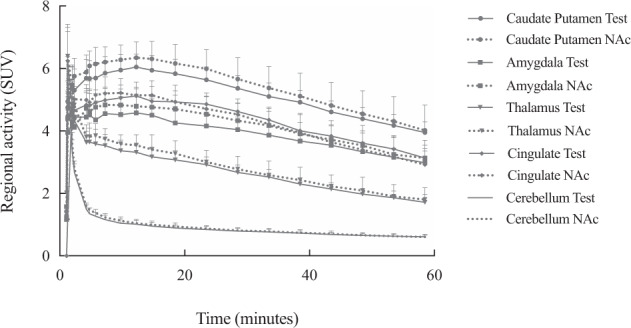
Time-activity curves for [^18^F]FPEB small animal PET for both conditions.

**Fig. 3:**
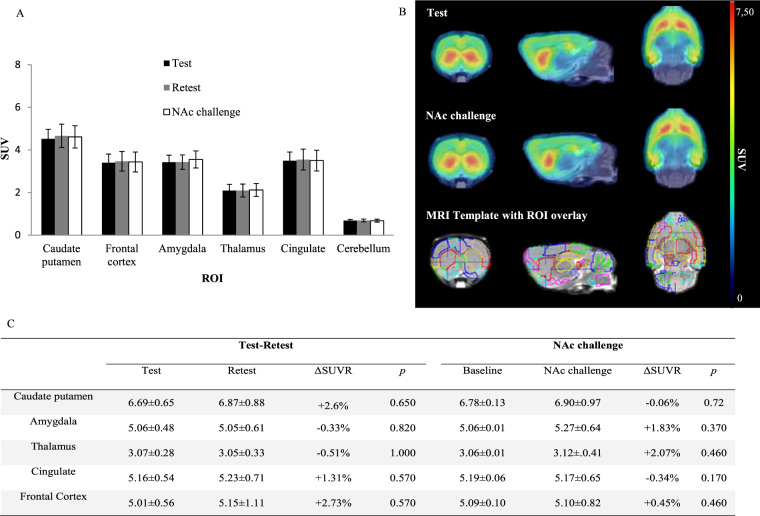
Preclinical study results. **A** [^18^F]FPEB SUV (mean ± SD) after test–retest and NAc challenge in six relevant regions of interest. **B** Illustrations of averaged [^18^F]FPEB SUV images after test and NAc challenge and VOI templates (PMOD) overlaid with [^18^F]FPEB template. **C** SUVr calculated with the cerebellum as the reference region and absolute ΔSUVR for test, retest, and NAc challenge in [^18^F]FPEB small animal PET (*n* = 9).

Mean time–activity curves (TAC) are presented in Fig. 2. After intravenous bolus injection of [^18^F]FPEB, a rapid uptake was observed in the 5 ROI examined (i.e., the caudate/putamen, amygdala, thalamus, cingulate, and cerebellum). After a peak (10 min post-injection), we observed that the maximum [^18^F]FPEB binding was on the striatum. Then it decreased slowly and linearly until the end of the acquisition. On the contrary, in the cerebellum, the uptake decreased sharply and remained low and stable from 20 min post-injection (p.i.). Figure 3A illustrates the averaged [^18^F]FPEB SUV calculated from the TAC for the different ROI and Fig. 3B illustrates microPET images of SUV as an overlay on a MRI template.

For both challenge conditions (test, retest, and NAc), we calculated the SUVr (SUV ratio to cerebellum) in five relevant brain areas (caudate putamen, amygdala, thalamus, cingulate, and frontal cortex) on small-animal PET as well as the absolute percentage changes across the baseline and NAc condition (Fig. 3C). SUVr values from test and retest (*n* = 9) were not significantly different in any region (*p* > 0.05). Similarly, Friedman two way analysis did not show any significant difference between SUVr values from baseline and from NAc challenge in any regions of interest (*p* > 0.05).

### Clinical MRS/PET study in healthy human subjects

**Fig. 4:**
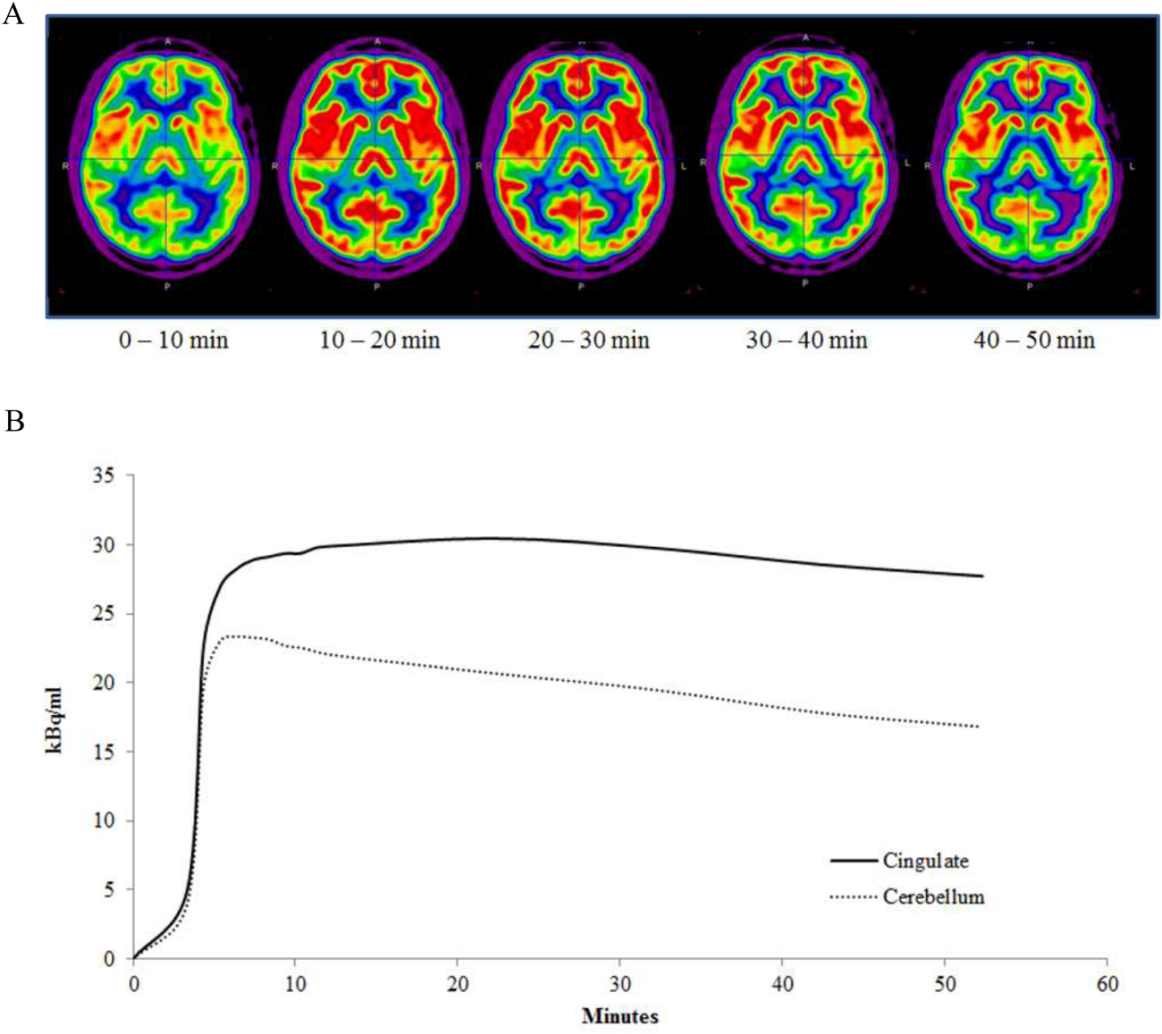
Clinical PET study results. **A** [^18^F]FPEB distribution after bolus injection in healthy volunteers. **B** Mean time–activity curves (kBq/mL) of [^18^F]FPEB in anterior cingulate cortex and cerebellum.

**Fig. 5:**
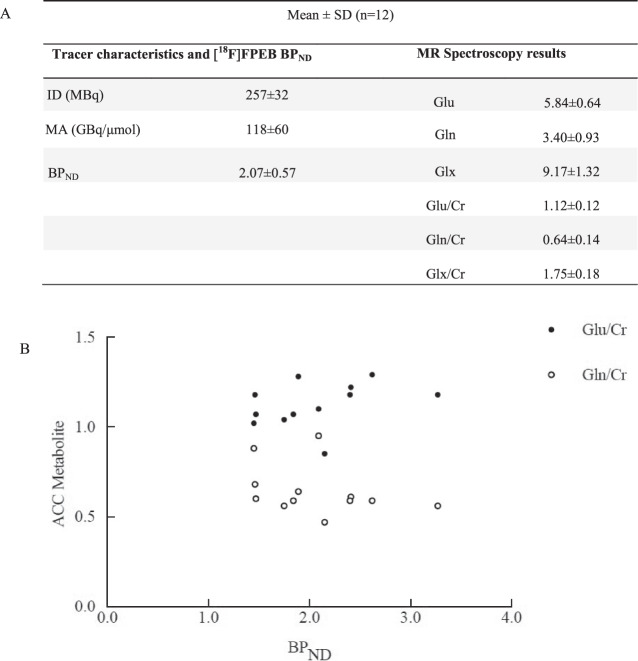
Clinical MRS/PET study comparison. **A** Tracer characteristics, [^18^F]FPEB non-displaceable binding potentials (BP_ND_), and MR spectroscopy results from the anterior cingulate cortex. **B** Scatter plots of the correlations between mGluR5 density and ACC levels of glutamate (Glu/Cr), glutamine (Gln/Cr) ratios in the ACC.

The mean age and weight of the study sample (*n* = 12 men) were 33 ± 8 years and 72 ± 13 kg, respectively. The regional brain mGluR5 availability in this sample was in agreement with the known distribution of mGluR5 in humans, with highest availability in the cingulate cortex (anterior and posterior), striatum and prefrontal regions and lowest availability in cerebellum and pons (Fig. 4). Then it decreased slowly and linearly until the end of the acquisition. We obtained good quality MR spectra from all subjects allowing reliable quantification. The mean glutamate and glutamine levels in the ACC were 5.84 ± 0.64 and 3.40 ± 0.93, respectively. Then, metabolites were quantified relative to creatine (Cr) and the mean Glu/Cr, Gln/Cr, and Glx/Cr levels were 1.12 ± 0.12, 0.64 ± 0.14, and 1.75 ± 0.18, respectively (Fig. 5). To test for an interaction of mGluR5 density and glutamate concentration, we correlated the [^18^F]FPEB BP_ND_ with Glu/Cr, Gln/Cr, and Glx/Cr ratios in the ACC. In the whole sample, ACC BP_ND_ did not correlated with ACC glutamate (*r* = 0.51; *p* = 0.09), glutamine (*r* = −0.46; *p* = 0.13) or Glx (*r* = −0.035; *p* = 0.92) (Fig. 5).

## Discussion

The purpose of the current study was to investigate by two complementary approaches whether [^18^F]FPEB, a PET ligand that binds to the allosteric site of the mGluR5, is sensitive to extrasynaptic glutamate concentration. Here, we investigated (i) the response of [^18^F]FPEB binding after a NAc pharmacological challenge in the rat brain, and (ii) the correlation between endogenous Glu, Gln and Glx levels measured by MRS and mGluR5 binding of [^18^F]FPEB in healthy human volunteers. Results indicate that the in vivo binding of [^18^F]FPEB to an allosteric site of the mGluR5 is not modulated by synaptic glutamate levels. Thus, [^18^F]FPEB appears unable to measure acute fluctuations in endogenous levels of glutamate in vivo. It could be assumed that the [^18^F]FPEB binding measures a certain stability of the mGluR5, which would not be influenced in the short term by fluctuations in glutamate concentrations.

Glutamatergic neurotransmission is a tightly regulated physiological process, involved in different brain functions. While glutamate is present at a millimolar concentration in both neuronal and astrocytic cells, the brain extracellular glutamate concentration is only 2–9 µM^49^. Indeed, vesicular glutamate is released into the synaptic cleft during the neurotransmission and is rapidly taken up by the astrocytic glutamate transporters (EAAT), where it is converted into glutamine by the glutamine synthetase. Glutamine is recycled into presynaptic neurons before being converted into glutamate by the glutamate synthetase. Non-vehicular free glutamate is released from astrocytes into the extracellular fluid via the system xc- that typically mediates the exchange of extracellular l-cystine and intracellular l-glutamate across the cellular plasma membrane^32^. Any changes in the extracellular glutamate concentrations, within the synaptic cleft, can be expected to modulate the activity of both metabotropic or ionotropic glutamate^50^. Extrasynaptically, glutamate released by the system xc- activates presynaptic mGluR2/3 and there, reduces the potential release of glutamate vesicles into the synaptic cleft. Postsynaptically, glutamate released by system xc- activates mGluR5^50^. PET is the only non-invasive imaging able to visualize, and quantify brain receptors in vivo under both physiological and pathophysiological conditions.

The cysteine pro-drug NAc can increase extracellular cystine, thereby it activates the glutamate release by the system xc- and subsequently increasing the signals through the presynaptic and postsynaptic mGluRs. The NAc dosage we used in the present preclinical study (50 mg/kg) was reported to induce significant increases in extracellular glutamate levels, as measured in vivo with microdialysis in the rat brain^51^. Our in vivo findings indicate that the increase levels of endogenous glutamate after the pharmacologic challenge with NAc did not interfere with [^18^F]FPEB binding to mGluR5 in the whole rat brain. The small-animal PET test–retest data indicated that the [^18^F]FPEB tracer bounds stably in delineated structures of the rat brain, such as the caudate putamen, amygdala, thalamus, cingulate, and cortex. Likewise, we did not observe significant changes of the [^18^F]FPEB time–activity curves in these cerebral areas.

In our PET/MRS imaging study, we report no significant correlation between glutamate metabolites levels and mGluR5 availability in vivo in the ACC in heathy subjects. This result is in agreement with Leurquin–Sterk et al.^29^. Indeed, considering that cerebral mGluR5 availability is associated with temperament traits in healthy humans, they found no relationship in healthy volunteers between MRS measures of glutamate in the ACC and personality measures or with regional [^18^F]FPEB binding. However the correlation between Glu and [^18^F]FPEB binding is closed to significance (*p* = 0.09), Regarding the numerous contradictory results (see below), whether with tracers or pharmacological molecules, it would be of interest to increase our sample size to address this issue. Our results support the hypothesis that [^18^F]FPEB, as well as [^11^C]ABP688 and [^18^F]PSS232, are modulators that bind to a site in the transmembrane domain and not to the orthosteric site where glutamate binds, and hence, their binding is supposed not to interfere with glutamate orthosteric binding.

A first [^18^F]FPEB challenge study, using ketamine, which acts as a potent glutamate antagonist on the NMDA receptor, has recently been reported in seven healthy subjects^41^. Holmes et al. suggested that [^18^F]FPEB may be less suitable than [^11^C]ABP688 for measuring changes in mGluR5 availability in drug challenge paradigms because the downregulation of mGluR5 at 24 h post-ketamine was not detected by [^18^F]FPEB, compared to [^11^C]ABP688.

Other mGluR5 radioligands have also been extensively explored using pharmacological challenges to assess their sensitivity to extrasynaptic endogenous glutamate concentration, at both preclinical and clinical levels. In accordance with our findings with [^18^F]FPEB in rats, the binding of [^11^C]ABP688 was not affected after the NAc challenge in both rats^39^ and Rhesus monkeys^40^. On the contrary, the ability of [^11^C]ABP688 to visualize acute fluctuations in endogenous glutamate levels was first suggested by Miyake et al. in a PET study performed in baboons^42^. In this study, the significant decrease in the tracer binding after NAc pharmacologic challenge suggests that a PET tracer targeting mGluR5 may be useful to measure acute in vivo changes in glutamate concentration. Recently, a clinical study using a simultaneous multimodal approach combining PET/MRS, has been carried out to assess acute changes in glutamate extracellular concentration after a stimulation with NAc, in ten healthy male adults^52^. This study conclude that, while MRS successfully detected acute fluctuations in glutamate and its metabolites concentrations in both basal ganglia and prefrontal cortex after NAc systemic administration, this effect was not found in PET imaging with [^18^F]PFSS232, suggesting that this radioligand was not sensitive to endogenous glutamate competition.

This approach based on pharmacological challenges has also given conflicting results using other glutamate modulators. A recent study in rats indicated that [^11^C]ABP688 was able to visualize acute fluctuations in endogenous glutamate levels after challenge with ceftriaxone, a potent GLT-1 activator, which induces a decrease of extracellular glutamate concentration^38^. In the other hand, Müller et al.^53^ concluded that [^18^F]PSS232 cannot measure in vivo fluctuations of glutamate levels induced with ceftriaxone in the rat brain.

Several authors have reported pharmacological challenges using ketamine, including two preclinical studies in rodents with [^11^C]ABP688^37^ and [^18^F]PSS232^54^ and two clinical trials, both performed with [^18^F]PSS232^55,56^. In rodent^37,54^, authors did not observed any significant changes in mGluR5 ligand binding from baseline to ketamine in any region, which confirms previous literature with other NMDA receptor antagonists in rodents. These data confirm previous results of Wyckhuys et al.^39^, and Sandiego et al.^40^, who also showed that [^11^C]ABP688 BP_ND_ did not reflect changes in acute endogenous glutamate fluctuations in rat and rhesus monkey, respectively, with other NMDA antagonists. However, these preclinical findings do not accord with those put forward by both clinical studies. De Lorenzo et al.^56^ found a significant reduction in [^11^C]ABP688 binding after ketamine administration as compared to baseline in the anterior cingulate, medial prefrontal cortex, orbital prefrontal cortex, ventral striatum, dorsal putamen, dorsal caudate, amygdala, and hippocampus. Thus, this study provided the first evidence that ketamine administration decreases the [^11^C]ABP688 binding in vivo in human subjects. Similar results have been also observed in Esterlis’s study in 2018^55^ using the same radiotracer, in 13 healthy and 13 major depressive disorder (MDD).

Thus, it appears difficult to conclude undoubtedly whether or not highly selective allosteric radioactive antagonists of mGluR5 are capable to image glutamate fluctuations. Overall, it seems that [^11^C]ABP688 is more sensitive to variations in endogenous glutamate than the other mGluR5 radioligands. The mechanism responsible for this change in [^11^C]ABP688 binding remains not clear and cannot be explained by direct competition. Interestingly, the exposure of most GPCRs such as mGluR5 to their agonizts results in an attenuation of responsiveness, or desensitization^57^. These regulatory mechanisms on mGluR in response to sustained high glutamate levels may contribute to mGluR5 internalization, leading to its unavailability to radioligands binding^28^. Furthermore, it also seems that this sensitivity of mGluR5 PET radioligands to endogenous fluctuations in glutamate is more frequently found in the human brain than in animals. In this context, our present study with [^18^F]FPEB appears to be of crucial importance, regarding its wide use in biomedical research in humans^27,28,30,58^.

Nevertheless, the present work has several limitations. First, the study samples were relatively small and clinical study included only men between 21 and 44 years old, which is not transposable to the general population that includes women and the elderly. Secondly, the MRS acquisition of the ACC voxel does not allow a measure of the entire ACC anatomical region, and this does not exclude that [^18^F]FPEB binding might be influenced by glutamate levels in other brain areas. Finally, ^1^H-MRS has significant limitations. The technique typically requires long-acquisition times, large voxels, low SNR, and low concentrations of the metabolites measured.

Our data suggest that [^18^F]FPEB is not sensitive to endogenous glutamate, whether in rodents with NAc, as in humans with ketamine^41^, or without a pharmacologic modulator i.e., in the physiological concentration ranges of glutamate. Thus, [^18^F]FPEB appears to be a robust tool to assess glutamatergic neurotransmission by PET and can allow a longitudinal assessment of the long-term expression of mGluR5.
